# Atypical teratoid/rhabdoid tumour with CDK6 amplification in a child: a case report and literature review

**DOI:** 10.3389/fped.2023.1237572

**Published:** 2023-08-22

**Authors:** Zhibin Li, Yubo Wang, Yuanhao Liu, Yining Jiang, Xuefei Han, Liyan Zhao, Yunqian Li

**Affiliations:** ^1^ Department of Neurosurgery, First Hospital of Jilin University, Changchun, China; ^2^ Department of Clinical Laboratory, Second Hospital of Jilin University, Changchun, China

**Keywords:** atypical teratoid/rhabdoid tumour, case report, central nervous system neoplasms, CDK6, treatment, CDK4/6 inhibitors

## Abstract

Atypical teratoid/rhabdoid tumours (AT/RTs) are rare central nervous system neoplasms that frequently occur in infants and children and have a very poor prognosis. In recent years, molecular analysis of AT/RTs has shown that biallelic inactivation of SMARCB1 (INI1, SNF5, BAF47) or SMARCA4 (BRG1) frequently occurs. Here, we present a case of basal ganglia AT/RT with SMARCB1 gene deficiency and CDK6 gene amplification in a 5-year-old child. A 5-year-old boy was hospitalized due to a 1-week history of frontal and parietal headache. Magnetic resonance imaging (MRI) demonstrated a 3 cm × 2 cm × 1.5 cm heterogeneous enhanced mass located at the right basal ganglia that partially protruded into the right lateral ventricle. The lesion was successfully resected under electrophysiological monitoring and neuronavigation. The postoperative pathological examination implied an AT/RT diagnosis, with loss of SMARCB1 protein, SMARCB1 gene deficiency and CDK6 gene amplification. Unfortunately, the patient died due to respiratory and circulatory failure at 5 weeks after the operation. To date, standard regimens have not yet been established due to the lack of large-scale prospective studies for AT/RT. The p16-RB signalling pathway should be considered as a potential target for AT/RT treatment modalities. Apart from traditional regimens, targeted therapies, especially CDK4/6 inhibitors, are likely a promising therapeutic option for AT/RT treatment.

## Introduction

Atypical teratoid/rhabdoid tumours (AT/RTs) are rare, aggressive, embryonal tumours of the central nervous system (CNS) that are characterized by rhabdoid cells and loss of SMARCB1 (INI1, SNF5, BAF47) or rare SMARCA4 (BRG1) protein (1–5). Rhabdoid tumours that occur in the CNS are defined as AT/RTs (6). They were initially reported in 1987 but recognized as a distinct tumour entity by the World Health Organization in 1993 (7, 8). The overall prognosis of AT/RT patients is poor, with a median survival time of approximately 17 months (9). AT/RT can occur in both infratentorial and supratentorial locations, but predominantly occurs in the posterior fossa, especially in the cerebellum and cerebellopontine angle (5, 10, 11). Biallelic SMARCB1 or rare SMARCA4 mutations, encoding core subunits of the SWI/SNF chromatin-remodelling complexes, are thought to be frequent genetic alterations in AT/RT (2, 12). Here, we present a case of AT/RT, which resulted in CDK6 gene amplification and, independently, SMARCB1 gene deficiency. Unfortunately, the patient died because of respiratory failure at 5 weeks after the operation. To the best of our knowledge, this study is the first report of a case of atypical teratoid/rhabdoid tumour with CDK6 amplification.

## Case report

**Figure 1:**
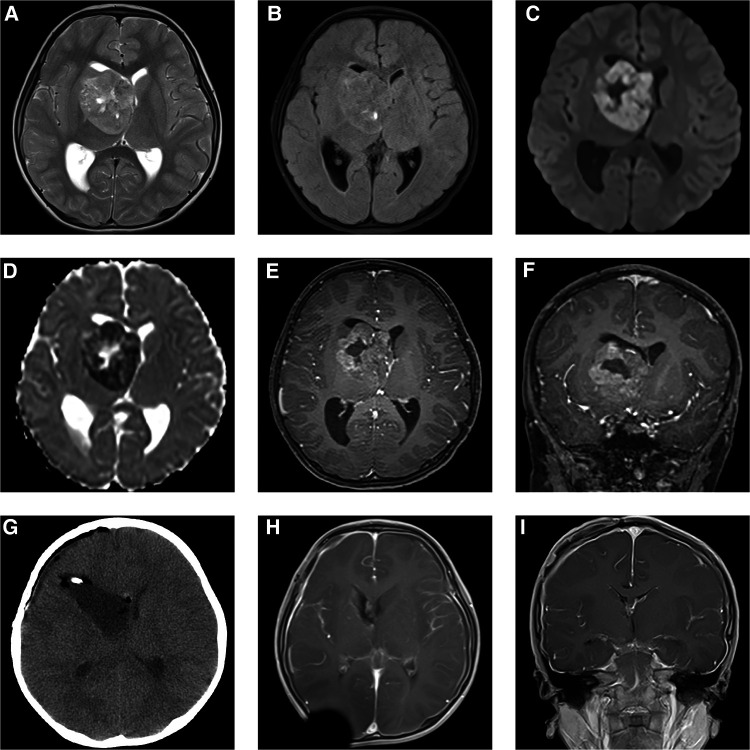
Preoperative MRI (**A–F**) demonstrated a 3 cm × 2 cm × 1.5 cm mass located in the right basal ganglia and thalamus, protruding into the right lateral ventricle. It was mixed isointense-to-hyperintense on T2WI (**A**) and T2 dark-fluid imaging (**B**). Hyperintense signals were shown by DWI (**C**), and inverse signals were indicated by the ADC (**D**). The tumour showed heterogeneous enhancement after gadolinium administration (**E,F**). Postoperative CT (**G**) and MRI 4 weeks after transfrontal-temporal craniotomy (**H,I**) suggested that the tumour was completely resected without signs of recurrence.

A 5-year-old boy was hospitalized due to a 1-week history of frontal and parietal intermittent headache. Based on physical examination, the right limbs were scored as a 5 and the left limbs as a 4 on the Lovett scale muscle grade. MRI showed a 3 cm × 2 cm × 1.5 cm large irregular cyst-solid lesion invading the right basal ganglia and thalamus and protruding into the right lateral ventricle. Uneven hypointense-to-isointense signals were observed on T1-weighted imaging (T1WI). Mixed isointense-to-hyperintense signals were shown on T2-weighted imaging (T2WI) (Figure 1A) and T2 dark-fluid imaging (Figure 1B), and hyperintense signals were observed on diffusion-weighted imaging (DWI) (Figure 1C); inverse signals were indicated by the apparent diffusion coefficient (ADC) (Figure 1D). After gadolinium administration, heterogeneous enhancement was observed, and a cyst was detected in the centre of the mass (Figures 1E–G).

A preoperative diagnosis of glioblastoma was made. However, the diagnosis of AT/RT could not be excluded. Transfrontal-temporal craniotomy was performed under preoperative and intraoperative neuronavigation and intraoperative electrophysiological monitoring of the supratentorial functional area. The tumour was pinkish, cyst-solid, and bloody. Postoperative MRI showed gross total resection (Figures 1H,I).

**Figure 2:**
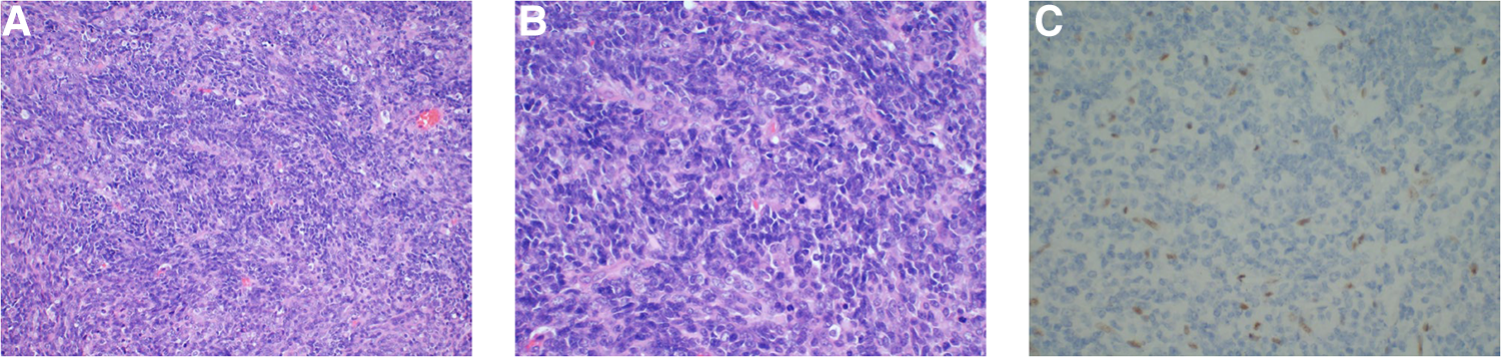
Haematoxylin and eosin-staining showed a large number of rhabdoid cells with eccentric nuclei, prominent nucleoli and eosinophilic cytoplasm (**A,B**). Immunohistochemical examination demonstrated INI-1 loss (**C**).

**Figure 3:**
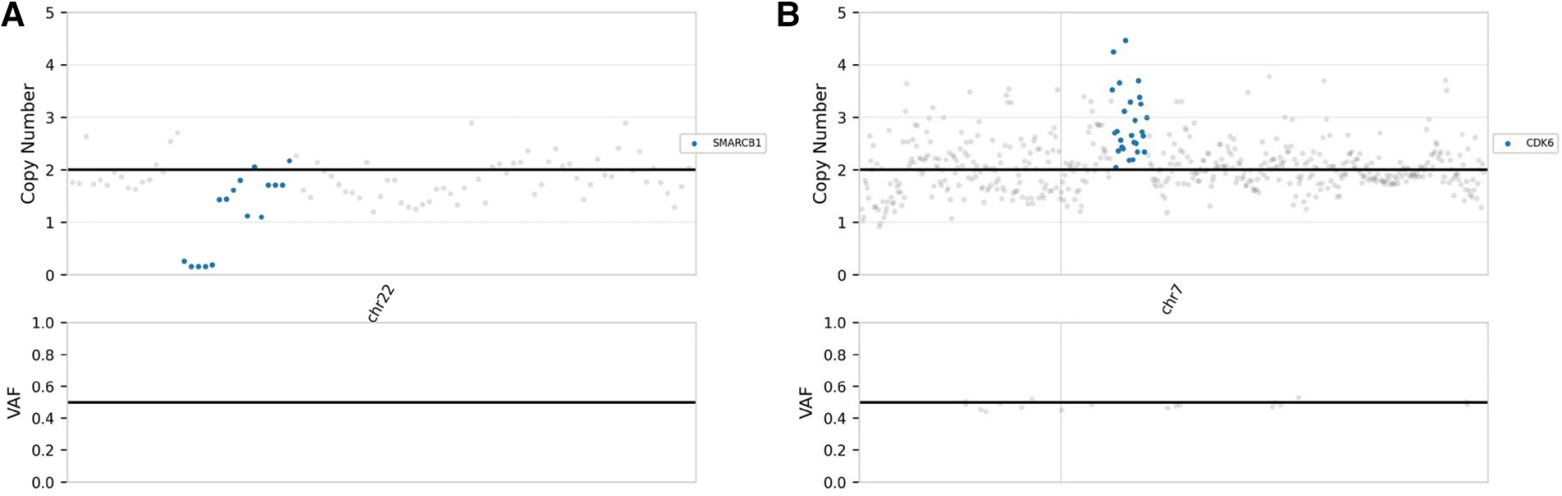
NGS demonstrated that the copy numbers of the SMARCB1 gene (**A**) and CDK6 gene (**B**) were 0.84 and 3.03, respectively.

The postoperative pathological analysis was suggestive of a diagnosis of AT/RT. Microscopic examination showed that the tumour comprised rhabdoid cells with eccentric nuclei, prominent nucleoli and eosinophilic cytoplasm (Figures 2A,B). Immunohistochemical examination indicated that the Ki-67 index was 80%, and INI-1 protein expression was deficient (Figure 2C). We also implemented next-generation sequencing (NGS) for diagnosis. Surprisingly, we not only found 2 categories of SMARCB1 gene mutations but also CDK6 gene amplification. One SMARCB1 gene mutation was exon 9 c.1130G > A p.R377H; the mutation abundance was 41.52%, which would dramatically reduce the combination of the C-terminal domain (CTD) of the SMARCB1 subunit, resulting in loss of functional proteins (13). SMARCB1 gene deficiency was also detected, and the copy number was 0.84 (Figure 3A). Moreover, we found that the CDK6 gene was amplified, with a copy number of 3.03 (Figure 3B). Integrating the histological, immunohistochemical and molecular examination, the AT/RT diagnosis was confirmed.

**Figure 4:**
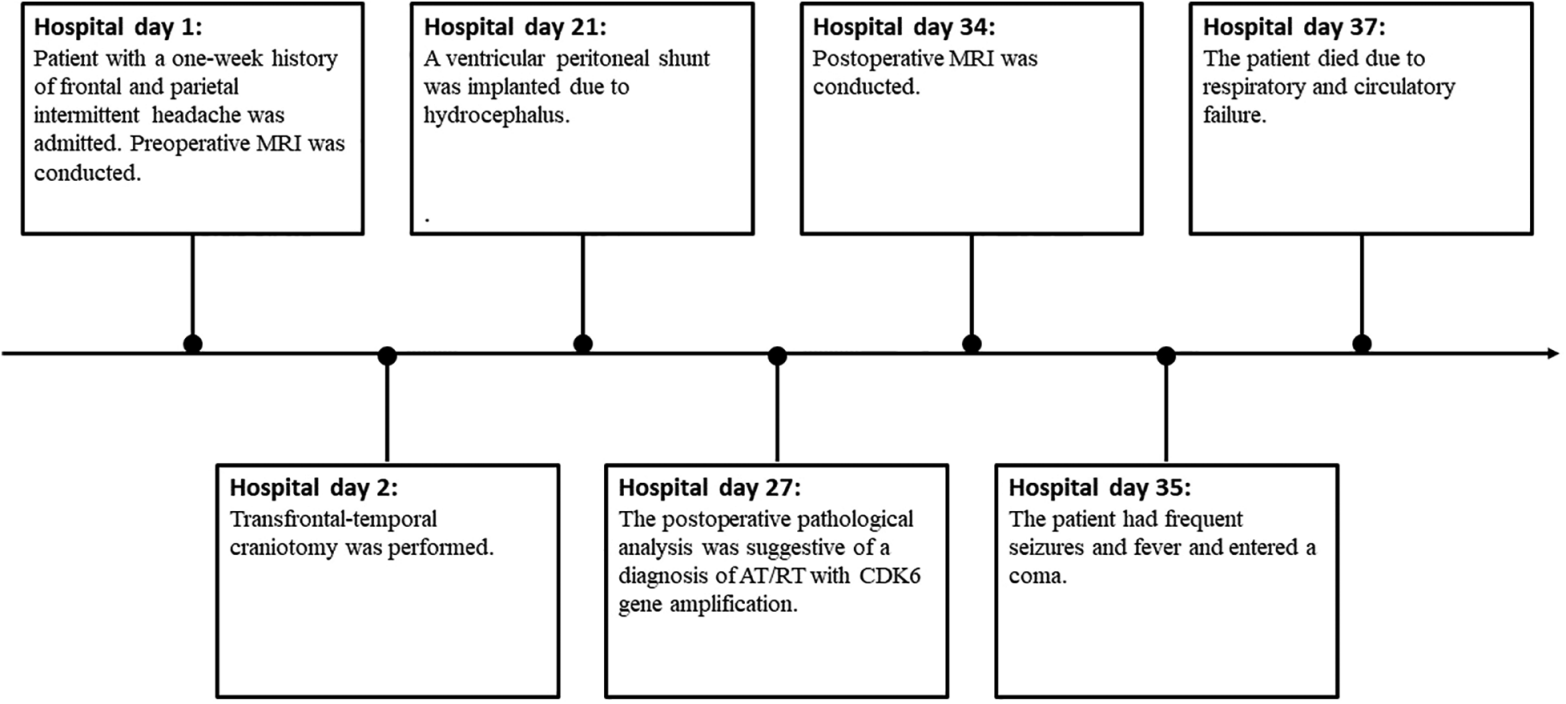
Timeline.

A ventricular peritoneal shunt was implanted at 19 days after transfrontal-temporal craniotomy because of hydrocephalus. Nevertheless, this patient had frequent seizures and fever and entered a coma. Unfortunately, the patient died owing to respiratory and circulatory failure arising from pulmonary infection and hydrocephalus 5 weeks after the operation (Figure 4).

## Discussion

The first time that a tumour entity was defined by molecular parameters other than histology was in the 2016 World Health Organization (WHO) classification of CNS tumours. The diagnosis of AT/RT required validation of specific molecular alterations, including inactivation of the SMARCB1 or SMARCA4 gene (14). Five years later, the fifth edition of the WHO classification of CNS tumours recognized 3 molecular subtypes of AT/RT, AT/RT-SHH, AT/RT-TYR and AT/RT-MYC, according to DNA methylation profiling and gene expression profiling. Using the WHO CNS tumour classification, the study by D'Arco et al. demonstrated that neuroimaging can help identify the tumour subtypes, providing information about prognosis and further management (15). Most AT/RTs demonstrate a hypointense signal on T1WI and variable signal on T2WI with heterogeneous contrast enhancement (15, 16). For one of the most important differential diagnoses, medulloblastoma, D'Arco et al. suggested that neuroimaging combined with clinical presentation could play an important role in differential diagnosis, as medulloblastoma occurred in an older age group than AT/RT (15). Other differential diagnoses should include choroid plexus tumours, other CNS embryonal tumours, high-grade gliomas, astrocytoma and ependymal tumours (17–19). For this patient, the preoperative diagnosis was glioblastoma. However, the diagnosis of AT/RT could not be excluded, and pathological examination indicated a diagnosis of AT/RT. To further confirm the diagnosis, we conducted NGS to validate the specific molecular alterations. Overall, integrating the clinical presentation, neuroimaging, pathological examination and NGS results, a diagnosis of AT/RT was made, which implied a very poor prognosis. After referring to the literature, we considered that selective CDK4/6 inhibitors might improve the prognosis of this child because of the CDK6 gene amplification detected by NGS. Unfortunately, the patient succumbed to respiratory and circulatory failure about 5 weeks after the operation, and the intervention of selective CDK4/6 inhibitors was not able to be administered.

AT/RT is a category of rare, aggressive, embryonal brain neoplasms. A study examining data from 13 European countries reported that the median age at AT/RT diagnosis is 29.5 months (20). The 5-year overall survival (OS) and event-free survival (EFS) are 34.7 ± 4.5% and 30.5 ± 4.2%, respectively (20). AT/RT comprises only 1%–2% of all paediatric CNS neoplasms but approximately 10%–20% of brain neoplasms in patients under 3 years old (21). Approximately 70% of cases occur in patients younger than 1 year old, with more than 90% in those younger than 3 years old (11).

Mutation of subunits of SWI/SNF chromatin-remodelling complexes is the central event in AT/RT. Studies in genetically engineered mouse models show that SMARCB1 is a genuine tumour suppressor gene (22–24). Biallelic mutations of SMARCB1 or SMARCA4 are frequent genetic alterations in AT/RTs (2, 25). The tumorigenesis of AT/RT follows a classical two-hit model (2). Approximately 35% of cases are identified as heterozygous germline mutations of SMARCB1 or SMARCA4, which constitutes the first hit (2). Another study demonstrated that germline mutations in AT/RTs can be identified at any age, and 60% of children under the age of 6 months are prone to multiple rhabdoid tumours (26). Therefore, genetic counselling is recommended for surveillance and identifying implications in pregnancies. In addition, the presence of germline mutations requires AT/RT patients to undergo whole-body examinations to detect potential extracranial lesions.

Due to the lack of large-scale prospective studies for AT/RT, a standard treatment regimen has not yet been established (27). Total tumour resection forms the typical basis for therapy (28–30). Although the implementation of radiotherapy in AT/RT patients remains controversial due to severe neurocognitive complications, the lack of alternative treatments has necessitated the continued use of radiotherapy as an important modality in AT/RT therapeutic regimens (27, 30). The efficacy of intensive chemotherapy and focal radiotherapy have been validated, with 37% 4-year EFS and 43% overall survival (31). This study, the first AT/RT-specific cooperative group trial, included 65 patients and identified high-dose chemotherapy with peripheral blood stem cell rescue (HDC/SCR) and involved-field radiotherapy as an important treatment modality for AT/RT.

SMARCB1 is implicated in many pathways, particularly the p16-RB pathway (32). The p16 tumour suppressor protein inhibits CDK4/6 (32). SMARCB1 is critical for regulating the expression of CDKN2A, which encodes p16 (33, 34). A principal consequence of SMARCB1 genetic inactivation is the inhibition of p16 expression, which correspondingly increases the activation of CDK4/6, facilitating the phosphorylation and inactivation of retinoblastoma tumour suppressor protein (RB) (35). Phosphorylated RB cannot suppress the E2F protein, which is required for progression from G1 to S phase, and thus, cell division is launched (32–34). The amplification or overexpression of CDK4/6 has been observed in several malignancies, including breast neoplasms, sarcoma, glioma, lymphoma and melanoma (36). In brain neoplasms, CDK6 amplification is frequently found in medulloblastoma glioma and glioblastoma (37–40). The NGS result of the present case is the first to show CDK6 amplification in AT/RT. The amplification of the CDK6 gene is related to high levels of CDK6 protein, which may increase the constitutive phosphorylation of RB, overriding p16 inhibition (41). Studies by Mendrzyk et al. and Ruano et al. suggested that CDK6 may promote the prognostic evaluation of medulloblastoma and glioblastoma, respectively (39, 40). This finding indicates that CDK6 may serve as a prognostic marker in AT/RT.

The use of CDK4/6 inhibitors palbociclib, ribociclib, and abemaciclib represents a major breakthrough in breast cancer therapeutics (42). More recently, CDK4/6 inhibitors have attracted attention as a potential option for AT/RT therapeutic regimens. Palbociclib is an oral selective inhibitor of CDK4/6, resulting in reduced RB phosphorylation and G1 phase arrest (43). A preclinical study by Hashizume et al. showed that palbociclib combined with irradiation therapy distinctly outperformed both therapies alone in terms of survival benefit to animal subjects. Furthermore, the study suggested an additional mechanism of palbociclib against tumours in which palbociclib expanded the period during which tumour cell irradiation impairs DNA double-strand breaks, supporting the application of palbociclib as a potential adjuvant to radiotherapy for AT/RT patients (35). Anderson et al. reported a case of asymptomatic nonsecreting pituitary adenoma and metastatic breast cancer treated with palbociclib and found regression of pituitary adenoma 1 year after initiating palbociclib (44). Although it is uncertain whether palbociclib contributes to the regression of pituitary adenoma, CDK6 was identified by another study by Zhang et al. as a potential therapeutic target of pituitary neuroendocrine tumours (45). In addition, palbociclib has been identified to be effective against several types of CNS neoplasms, including glioma, glioblastoma, medulloblastoma and breast cancer brain metastases (35, 46–48). Ribociclib is another CDK4/6 inhibitor, resembling palbociclib (49). A phase I study by Geoerger et al. on malignant rhabdoid tumours revealed prolonged stable disease in paediatric patients with an oral single-agent ribociclib regimen (50). One other phase 0 trial suggested that ribociclib demonstrated good CNS penetration across not only a disrupted blood-brain barrier (BBB) but also an intact BBB in human glioblastoma regions, which is a promising result and encourages us to explore ribociclib for treating AT/RT (49). Overall, selective CDK4/6 inhibitors may improve the prognosis of AT/RT patients. Palbociclib and ribociclib may represent promising molecular agents against AT/RT, especially for the present case with CDK6 gene amplification. Nevertheless, further clinical trials should be conducted to determine the validity of these agents for AT/RT treatment and their permeability of the BBB.

## Conclusion

Since AT/RT is a category of CNS neoplasm with very poor prognosis, long-term follow-up, prospective studies and clinical trials are warranted to fully understand this disease. We highlighted the function of CDK4/6 in the tumorigenesis of AT/RT and the importance of CDK4/6 inhibitors as a potential option for AT/RT therapeutic regimens, serving as a potential guide for clinicians and clinical studies.

## Data Availability

The datasets presented in this study can be found in online repositories. The names of the repository/repositories and accession number(s) can be found below: https://www.ncbi.nlm.nih.gov/sra, PRJNA936291.
